# dIvergEnt: How IgE Axis Contributes to the *Continuum* of Allergic Asthma and Anti-IgE Therapies

**DOI:** 10.3390/ijms18061328

**Published:** 2017-06-21

**Authors:** Óscar Palomares, Silvia Sánchez-Ramón, Ignacio Dávila, Luis Prieto, Luis Pérez de Llano, Marta Lleonart, Christian Domingo, Antonio Nieto

**Affiliations:** 1 Department of Biochemistry and Molecular Biology, School of Chemistry, Complutense University of Madrid, 28040 Madrid, Spain; oscar.palomares@quim.ucm.es; 2 Department of Clinical Immunology and Health Research Institute of the Hospital Clínico San Carlos (IdISSC), Hospital Clínico San Carlos, 28040 Madrid, Spain; ssramon@salud.madrid.org; 3 Department of Microbiology I, Complutense University School of Medicine, 28040 Madrid, Spain; 4 Allergy Service, University Hospital of Salamanca and Institute for Biomedical Research of Salamanca (IBSAL), Biomedical and Diagnosis Science Department, Salamanca University School of Medicine, 37008 Salamanca, Spain; idg@usal.es; 5 Department of Allergy and Immunology, University of Valencia and Dr. Peset University Hospital, 46017 Valencia, Spain; prieto_jes@gva.es; 6 Neumology Service, Hospital Universitario Lucus Augusti, 27003 Lugo, Spain; eremos26@hotmail.com; 7 Novartis Farmacéutica, 08013 Barcelona, Spain; marta.lleonart@novartis.com; 8 Pulmonary Service, Corporació Sanitària Parc Taulí, Department of Medicine, Universitat Autònoma de Barcelona (UAB), 08193 Barcelona, Spain; 9 Pediatric Pulmonology & Allergy Unit, Children’s Hospital La Fe, 46026 Valencia, Spain; nieto_ant@gva.es

**Keywords:** allergy, asthma, immunoglobulin E (IgE), biomarkers, immunological mechanisms, immunomodulation, biological treatment, anti-IgE, omalizumab

## Abstract

Asthma is an airway disease characterised by chronic inflammation with intermittent or permanent symptoms including wheezing, shortness of breath, chest tightness, and cough, which vary in terms of their occurrence, frequency, and intensity. The most common associated feature in the airways of patients with asthma is airway inflammation. In recent decades, efforts have been made to characterise the heterogeneous clinical nature of asthma. The interest in improving the definitions of asthma phenotypes and endotypes is growing, although these classifications do not always correlate with prognosis nor are always appropriate therapeutic approaches. Attempts have been made to identify the most relevant molecular and cellular biomarkers underlying the immunopathophysiological mechanisms of the disease. For almost 50 years, immunoglobulin E (IgE) has been identified as a central factor in allergic asthma, due to its allergen-specific nature. Many of the mechanisms of the inflammatory cascade underlying allergic asthma have already been elucidated, and IgE has been shown to play a fundamental role in the triggering, development, and chronicity of the inflammatory responses within the disease. Blocking IgE with monoclonal antibodies such as omalizumab have demonstrated their efficacy, effectiveness, and safety in treating allergic asthma. A better understanding of the multiple contributions of IgE to the inflammatory continuum of asthma could contribute to the development of novel therapeutic strategies for the disease.

## 1. Introduction

Asthma is one of the world´s most common chronic airway diseases, characterised by recurrent symptoms associated with variable airflow obstruction, bronchial hyperresponsiveness (BHR), and inflammation [1,2]. The term asthma encompasses a syndromic definition including different clinical phenotypes and pathophysiological pathways, yielding a complex clinical scenario with several disease variant classifications [3,4]. Many subtypes of asthma have been described on the basis of different clinical phenotype definitions associated with different triggers [4,5,6,7]. For its part, the endotype classification includes different etiological and pathophysiological mechanisms, thus allowing identifiable gene-expression profiles and biomarkers for the design of new therapeutic strategies [3,8]. Wenzel [4] emphasised that although several endotype classifications of asthma have been proposed, none have been met with wide-ranging agreement.

The prevalence of allergic diseases has increased all over the world in recent decades [9,10,11,12], and in fact these conditions have become a real pandemic phenomenon [13]. On average, around 10–12% of children under the age of 6 or 7 years, 14% of adolescents aged between 7 and 14 years, and 5% of the global population suffer from asthma, and more than 250,000 annual deaths are attributed to the disease [14,15]. Up to 90% of childhood asthma is allergic, and children with allergies have a 30% increased risk of developing asthma [16]. After following a cohort of 1041 children with mild-moderate asthma, the Childhood Asthma Management Program (CAMP) reported that asthma appears to be periodic in 39% of cases and persistent in 55%, and that no effect of earlier anti-inflammatory treatment was noted [17,18,19]. The CAMP study emphasised that children are exposed to a wide variety of environmental factors which are known to trigger symptoms, despite attempts to modify the environment in their homes [20].

Asthma affects patients of all ages and represents a serious public health problem with a high socioeconomic impact. In Europe, annual care costs (direct and indirect) of persistent asthma in the whole of the European population aged from 15 to 64 years exceed EUR 19 billion [21]. Therefore, in order to achieve prompt control, there is an urgent need to determine the factors directly associated with the disease. The search for new therapeutic options should focus on clearly stated key molecules.

Most cases of asthma are due to an immunoglobulin E (IgE)-mediated reaction after sensitization to inhaled allergens. IgE belongs to the Ig family (Table 1), proteins that bind to the specific antigens which are used by the immune system to protect an organism against pathogens. Allergic asthma is associated with increased levels of circulating total and specific IgE, with a clear involvement both at the onset of the disease and during its chronic phase. Thus, IgE has emerged as the most promising target for the management of the allergic form of the condition [22]. In this review, we discuss the central role of IgE-mediated pathophysiological and inflammatory mechanisms in all the phases of allergic asthma.

## 2. The Iceberg Model of Allergic Asthma: Immunological Pathways beyond Visible Clinical Symptoms

**Figure 1:**
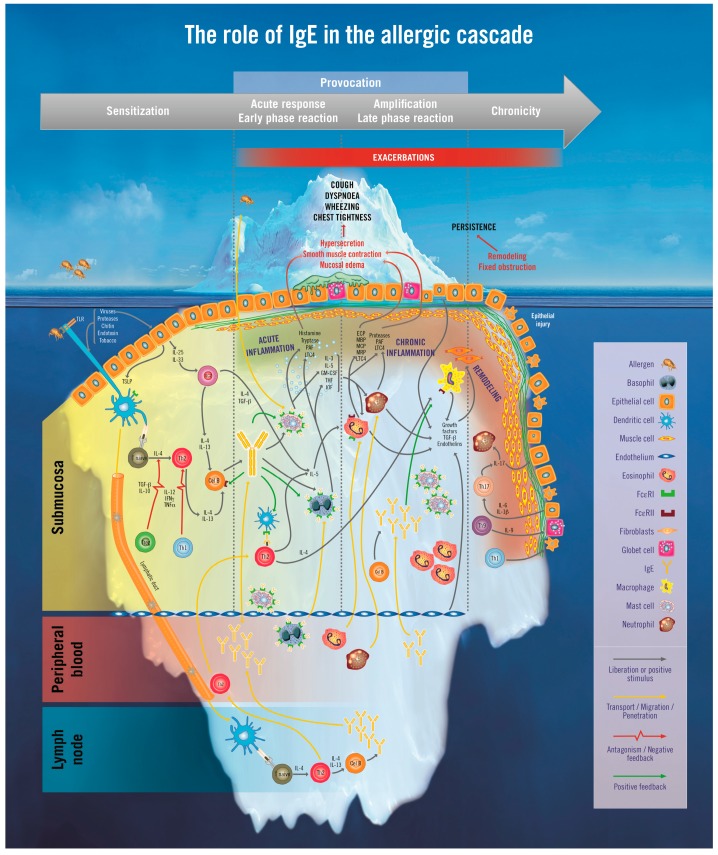
The Iceberg model of allergic asthma. Allergic asthma is characterised by a visible part of the disease; however, many pathophysiological changes of this complex process occur in the depths. Changes at the level of lymphatic nodes, peripheral blood, and submucosa appear from the beginning of the disease and should be addressed in order to minimise the impact and persistency of symptoms. The influence of IgE is present across all levels of the iceberg. Further details can be found in the text. ECP: eosinophil cationic protein, FcεRI: high affinity IgE receptor, FcεRII: low affinity IgE receptor, GM-CSF: granulocyte-macrophage colony-stimulating factor, IFNγ: interferon gamma, IgE: immunoglobulin E, IL: interleukin, ILC: innate lymphoid cells, LTC4: leukotriene C4, MBP: major basic protein, MCP: monocyte chemotactic protein, MRP: myeloid related proteins, PAF: platelet-activating factor, TGFβ: transforming growth factor beta, Th: T helper cells, TLR: toll-like receptors, TNFα: tumor necrosis factor alpha, Treg: regulatory T cells, TSLP: thymic stromal lymphopoietin.

If we compare asthma with an iceberg, clinical symptoms such as cough, wheezing, breathlessness, and chest tightness, which can be directly observed by clinicians, would be the equivalent to the tip of the iceberg. However, to explain the complex process of asthma that triggers clinical symptoms we need to explore the iceberg in depth (Figure 1).

A patient with allergic asthma may clinically manifest the aforementioned symptoms after exposure to a specific environmental allergen to which he or she is sensitised, and may suffer later exacerbations. Some patients show persistent symptoms over time. As previously mentioned, most asthmatic children present an allergic component [29,30], and the presence of allergen-specific IgE has been shown to predict later asthma development [31]; this allergic phenomenon correlates with asthma severity in different age ranges and populations [32,33,34,35]. Allergic sensitization is identified in 86% of adults with asthma onset before the age of 6 years and in 49% of young adults aged between 22 and 40 years [36]. Asthma severity, poor asthma control, and recurrent exacerbations all correlate with high serum IgE levels [35,37,38,39]. Hospitalisations and deterioration in lung function have also been associated with high total serum IgE [35]. Several studies of IgE-mediated persistent severe allergic asthma suggest that the disease is better controlled under a treatment that targets the IgE pathway [40,41,42].

## 3. The Role of Immunoglobulin E (IgE) in Allergic Asthma

The understanding of the immunological mechanisms underlying allergic asthma has advanced significantly in recent decades. Today, it is accepted that there are two consecutive, well-defined stages: the sensitization phase and the re-exposure phase. The re-exposure phase comprises two types of event: early acute and late-phase reactions. Perpetuation of the inflammation may also contribute to the chronic phase, which is associated with more severe clinical manifestations of asthma, including frequent exacerbations as well as irreversible airway damage.

### 3.1. The Sensitization Phase

The immune allergic response begins with sensitization (Figure 1). This phase occurs when the patient is first exposed to an allergen. At this stage, there are no clinical symptoms. Dendritic cells (DC), which are the main allergen-presenting cells (APC) located in the respiratory tract, capture the allergen and process it while migrating to the nearest lymph node. The processed allergen is then presented to naïve CD4+ T lymphocytes in the context of major histocompatibility complex (MHC)-class II-associated peptides. These T cells are activated and differentiated into allergen-specific T helper type 2 (Th2) cells (a process which is partially dependent on interleukin IL-4) [43,44]. Allergen-specific Th2 cells collaborate in the activation and isotype-switching of B cells to produce high levels of allergen-specific IgE antibodies. Once IgE is released into the circulation, it binds through its Cε3 domain to high affinity IgE receptor (FcεRI) and CD23 located on the surface of effector cells, such as mast cells (MC) and basophils and other relevant cells in the pathophysiology of asthma, such as DC, monocytes, and smooth muscle cells (SMC).

During this phase, a pool of memory allergen-specific Th2 and B cells that will play an important role in the subsequent phases is generated. Innate non-hematopoietic immunity is also present during the sensitization phase, especially with the involvement of the epithelial cells. Classically, the bronchial epithelium has been considered a merely physical barrier protecting the internal milieu against foreign aggressions. Today, it is known that it is able to respond to several external stimuli (allergens, viruses, proteases), triggering the production of Th2-promoting cytokines, such as thymic stromal lymphopoietin, IL-25 or IL-33 [45], which activate DC, favouring the generation of type 2 responses and the further production of IgE. Other cells, such as basophils, natural killer T cells or type 2 innate lymphoid cells are also important in the sensitization phase, producing different cytokines (such as IL-4) and amplifying Th2 responses.

B cells can also act as APCs, triggering Th2 differentiation and generating IgE-specific antibodies. Memory B cells differentiate into short-lived plasma cells or to allergen-specific long-lived plasma cells, which remain in the bone marrow. The IgE repertoire of a given patient can include specific low-affinity IgE that can also trigger the sensitization phase [46]. Recently, a study has used high-throughput DNA sequencing of Ig heavy chain rearrangements to identify clonal lineages of B cells containing members expressing IgE [47]. The results demonstrate that the primary source of IgE in humans is the secondary isotype switching of IgG1-expressing B cells. Thus, this suggests that IgE is derived from antigen-experienced B cells rather than from naive B cells with low-affinity, non-mutated antibodies. These data provide a basis from which to evaluate allergen-specific lineages with IgE members that are responsible for human allergic disease [47].

### 3.2. The Re-Exposure Phase

#### 3.2.1. The Acute Reaction

Upon re-exposure, the causative allergen cross-links to at least two IgE molecules bound to FcεRI on MC or basophils, with a subsequent release of diverse preformed mediators (histamine, heparin, TNF-α) and newly synthesised lipid mediators (prostaglandins and leukotrienes). At this point, an IgE-mediated type I early hypersensitivity reaction can occur within minutes of allergen exposure. These events may cause responses at a local level, such as bronchoconstriction, vasodilation, and/or airway mucus secretion. These pathophysiological events trigger the allergy-associated symptoms of nasal congestion, wheezing, sneezing, cough, conjunctivitis, runny nose, dyspnoea, and chest tightness.

#### 3.2.2. The Late-Phase Response

Usually preceded by a clinically evident early-phase reaction, the late-phase response may produce delayed and more persistent effects. It has been described in approximately 50% of adult allergic asthmatic patients upon allergen challenge [48,49]. The late-phase reaction occurs after the activation of memory allergen-specific Th2 cells by APC (DC and B cells) through a process partially dependent on IgE-facilitated presentation and due to the accumulation of MC-derived mediators in the exposed local tissues. Activated allergen-specific Th2 cells and MC produce different mediators, cytokines, and chemokines that drive the infiltration and activation of inflammatory cells such as eosinophils, memory Th2 cells, neutrophils, or basophils to the lung parenchyma.

Memory allergen-specific Th2 cells previously activated by IgE-facilitated presentation produce a plethora of cytokines (IL-5, IL-13, IL-4 or IL-9), chemokines, and adhesion molecules that contribute to the recruitment of eosinophils and to the activation of effector cells, which release a series of mediators and toxic proteins (eosinophilic cationic protein, major basic protein, leukotrienes, etc.). These are responsible for the typical pathophysiological findings of the late phases of allergic asthma: increased vascular permeability, bronchial oedema, bronchospasm, and hypercrinia/dyscrinia.

Then, inflammatory cells (eosinophils) infiltrate the airways. This process is mainly mediated by chemokines and IL-5 produced by allergen-specific memory Th2 cells. Th2 cells and Th2-derived cytokines also contribute to the local production of IgE by B cells, airway muscle contraction, and increased vascular permeability favouring inflammation and exacerbated mucus production by goblet cells, all of which leads to bronchial hyperresponsiveness and obstruction, characteristic of the late severe phases of allergic asthma. Other cells that can also contribute to the exacerbation of these phenomena include epithelial cells, type 2 innate lymphoid cells producing Th2 cytokines, natural killer T cells, and Th1 or Th17 cells.

#### 3.2.3. The Chronic Phase

The late response can evolve into a chronic inflammatory response, which may be induced by repetitive exposure to the specific allergen that stimulates allergen-specific Th2 cells and MC, which in turn promote more eosinophilia and additional IgE production. This chronic phase typically involves the presence not only of large numbers of innate and adaptive immune cells at the affected site, but also the presence of changes in the extracellular space and alterations in the number, phenotype, and function of structural cells in the affected tissues. In this regard, apart from its indirect effect on the perpetuation of the inflammation, IgE may also play an important role in the airway remodelling, as evidenced by recent studies which have suggested a direct effect of this immunoglobulin in airway structural changes [50,51]. Persistent inflammation may promote the generation of airway remodelling, leading to a progressive loss of lung function and fixed airway obstruction.

## 4. Immunopathophysiology of IgE in the Asthma Continuum

**Figure 2:**
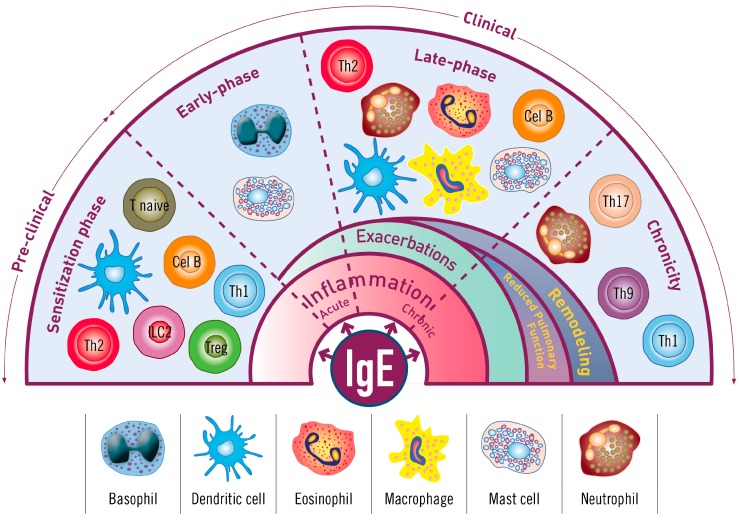
A continuum scenario in the immunoglobulin E (IgE) role in allergic asthma. The IgE has a central role in the continuum cascade of allergic reaction and participates in all phases: the sensitivity phase reaction, the early clinical phase, the late clinical phase, and the final chronic consequences. ILC2: type 2 innate lymphoid cells, Th: T helper cells, Treg: regulatory T cells.

Although the development of allergic reactions is characterised by a wide heterogeneity, IgE plays a crucial role in bridging innate and adaptive immunopathological events in the continuum of allergic asthma, from allergic sensitization to clinical early and late phases, and its evolution into a chronic condition, by acting on different immune cells and modifying their functions (Figure 2).

IgE exerts important immunomodulatory effects by binding to its high-affinity and low-affinity receptors. The FcεRII is constitutively expressed in different cells, helping to perpetuate the allergic response. When IgE links to its receptors on the effector cells, even in the absence of an allergen, there is an induction of the receptor expression, a cytokinergic effect, and a stabilization of the receptors [52]. This increases the viability of MC and can induce the cascade of proinflammatory cytokines in contact with the allergen [52,53]. MC have a central role in the initiation of the allergic immune response, providing signals that induce and/or maintain IgE synthesis as well as Th2 lymphocyte differentiation [54]. Recent findings have also indicated that MC have immunomodulatory properties [55]. An ongoing IgE-dependent activation of MC may contribute to the increase in vascular damage, infiltration by inflammatory cells, and to the increase in the migration and maturation of DC [56,57]. The consequence may be an increase in BHR and airway remodelling which appears to be strongly associated with the persistence of asthma.

For their part, Th2 cytokines have been considered as central mediators in allergic asthma, operating through mechanisms other than those classically implicated in allergic responses [58]. Cytokines act on the bronchial epithelium, the mucosa and bronchial SMC, producing BHR and clinical manifestations of asthma. In some patients, this process becomes chronic, with IgE and MC as the initial drivers of the long-term pathophysiological changes and tissue remodelling associated with chronic allergic reactions [59].

So, with the emerging knowledge on the role of IgE as a key factor in the pathophysiological process of allergic asthma, its therapeutic potential and relevance need to be reassessed.

## 5. Should IgE Blocking Be a Key Therapeutic Target for Allergic Asthma?

The idea that asthma is a chronic inflammatory airway disease has been the rationale for the treatment with anti-inflammatory agents, namely inhaled corticosteroids. However, assuming that inflammation is a consequence rather than the cause of the problem, treating allergic asthma with pleiotropic anti-inflammatory drugs alone would imply treating exclusively the tip of the iceberg. This approach has shown to be helpful to palliate symptoms, but this treatment does not modify the nature of the disease (Figure 1).

In this scenario, a novel therapeutic approach to asthma and other allergic respiratory diseases focuses on interfering with the effects of IgE [60]. Omalizumab is an anti-IgE humanised monoclonal antibody which inhibits IgE effector function by binding to free IgE at the same site as FcεRI, and thereby impedes IgE binding to FcεRI on effector cells, thus avoiding MC and basophil activation [61,62,63]. Initial studies of omalizumab in patients with mild asthma showed that blocking IgE reduced the early bronchoconstriction response to inhaled allergens. Furthermore, omalizumab has been shown to decrease eosinophilia in blood [64,65]. Omalizumab was approved for use in asthma and has been used for more than 10 years now, with extensive reports of evident clinical benefits in the literature [66]. Moreover, omalizumab has helped to identify additional roles of IgE in allergic asthma beyond the suppression and blocking of the immediate allergic reaction [67,68].

Biological modification of Th2-type cells has been also reported as an approach for specific patients in whom the appropriate Th2 immune pathway is predominant [67,69,70]. Table 2 summarises some conceptual aspects that distinguish between the approaches of blocking Th2 cytokines and the IgE, which are not mutually exclusive.

Recent findings suggest that, under certain conditions, treatment with anti-cytokine monoclonal antibodies can potentiate the target cytokine rather than neutralise its activity [71]. This is likely due to the formation of cytokine/anti-cytokine complexes, which might explain why targeting cytokines could be clinically inefficient if the employed doses of the monoclonal antibody are low enough to favour the formation of these immune complexes [71]. It should be also borne in mind, in any case, that IgE blockade also indirectly inhibits the production of Th2 cytokines by memory allergen-specific Th2 cells and MC, thus contributing to inhibit acute early responses, reducing inflammation and maintaining homeostasis [72,73,74].

The clinical and immunological benefits of blocking IgE include: (i) the prevention of IgE fixation to high-affinity receptors in MC and basophils, thus avoiding the release of mediators after allergen linkage; (ii) the reduction of basophils [75] and MC survival; (iii) the decrease in local IgE production; (iv) the blockade of total circulating IgE and IgE located in the lymph nodes; (v) the disabling of IgE-facilitated allergen presentation (DC and Th2 lymphocytes) [76]; (vi) the decrease in the release of IL-4 [72] and IL-5 [73] and consequently in their concentration levels; and (vii) an additional antiviral effect by upregulating the expression of interferons by plasmacytoid DC through a reduction of the crosslinking of IgE with the DC FcεRI [77,78]. This latter feature may have a clinical impact on viral-induced asthma exacerbations, which are very frequent in children [77,78,79,80]. Bearing all these aspects in mind, the final outcome of anti-IgE treatment is an attenuation of most of the acute and late responses, together with a lower risk of exacerbations observed in patients with allergic asthma. Thus, it appears that the modulation of IgE is of paramount importance for the successful treatment of allergic asthma.

Numerous clinical trials and real-life studies have demonstrated anti-IgE (omalizumab) as a successful treatment to reduce exacerbations, hospitalisations, visits to specialists, and medication use as well as to improve symptom control and quality of life in severe asthma patients [66]. Other biologicals targeting type 2 cytokines or their receptors have been also recently approved or are under development as add on therapies for severe asthma, such as anti-IL5 monoclonal antibodies (mAbs), anti-IL5Rα, or anti-IL4Rα mAbs [81,82,83,84,85,86,87]. As above discussed, due to the central role of IgE in the allergic inflammatory pathways, anti-IgE treatments have been also shown to partially impair the production of Th2 cytokines such as IL-5, IL-4, and IL-13. However, up to date, head-to-head comparative studies for all these biological agents have not been reported and future research will help to elucidate which severe asthma specific phenotypes/endotypes might better benefit from each specific biological treatment.

## 6. Conclusions

Allergic asthma is a heterogeneous airway disease triggered by the exposure of the patient to environmental allergens. Traditionally, asthma and allergic diseases have been broadly defined and treated with non-specific anti-inflammatory drugs and bronchodilators. With the recognition of allergic asthma as an allergen-specific disease with heterogeneous phenomena, together with the recent cluster analysis definition, and molecular and clinical phenotyping, a more targeted therapy may open up new avenues for the treatment of allergic asthma.

IgE plays a central role from the very start of the disease and throughout its continuum. Controlled clinical trials and real-life studies carried out over more than 10 years have demonstrated that IgE blocking shows a notable profile of effectiveness, efficacy, and safety in the treatment of moderate to severe allergic asthma [88,89,90,91,92]. Blocking the IgE axis appears to have a series of effects beyond its expected mechanistic action, as new information concerning the roles of IgE in the pathophysiology of the allergic asthma comes to light. Future studies should assess the potential long-term disease-modifying effect of anti-IgE therapeutic strategies. Additional efforts should also be made to discover novel biomarkers and potential targets for improving the field of personalised and precise therapeutic strategies in order to prevent allergic asthma from becoming chronic.

## Figures and Tables

**Table 1:** Summary of human immunoglobin and their main function.

Name	Subclasses	Form	Location	Main Function
IgA	2	Monomer, dimer and polymer	Mucosal tissue and blood	Opsonization and immune exclusion by binding to noxious antigens and preventing the adherence of microorganisms to the surface epithelium [23]
IgD	1	Monomer	Surface of mature B cells and blood	Transmembrane antigen receptor of unexposed antigen to complement the functions of IgM [24,25]
IgE	1	Monomer	Blood	To mediate the signalling response to pathogens [26]
IgG	4	Monomer	Blood	To bind to antigens to mediate the signalling response to antigens [27]
IgM	1	Monomer and pentamer	Surface of mature B cells and blood	Initial response to infections [28]

**Table 2:** Physiological differences between immunoglobulin E (IgE) and T helper type 2 lymphocytes (Th2) cytokines.

IgE	Th2 Cytokines
Type I (and also IV) hypersensitivity	Type IV hypersensitivity (IVb)
Recognition and specific memory for the involved allergens (e.g., venoms, environmental irritants)	No memory
Control in the effector arm of the allergy	Chemical messengers
Local and systemic effects	Local effects
Mean half-life 2.5 days (months when linked to its receptor)	Mean half-life, minutes
Central axis of Th2 response	Redundancy in asthma
Beneficial physiological role: response against helminthes	Beneficial physiological role: tissue repair and response against extracellular organisms
Examples of hypersensitivity reaction: allergic rhinitis, asthma, systemic anaphylaxis	Examples of hypersensitivity reaction: chronic asthma, chronic allergic rhinitis

## Abbreviations

APC	Allergen-presenting cells
BHR	Bronchial hyperresponsiveness
FcεRI	High affinity IgE receptor
FcεRII	Low affinity IgE receptor
FEV1	Forced expiratory volume in 1 second
FVC	Forced vital capacity
IgE	Immunoglobulin E
IL	Interleukin
IS	Immune system
MC	Mast cells
SMC	Smooth muscle cells
Th2	T helper type 2 lymphocytes

## References

[1] Martinez F.D., Vercelli D. (2013). Asthma. Lancet.

[2] Bel E.H. (2013). Clinical Practice. Mild asthma. N. Engl. J. Med..

[3] Xie M., Wenzel S.E. (2013). A global perspective in asthma: From phenotype to endotype. Chin. Med. J..

[4] Wenzel S.E. (2012). Asthma phenotypes: The evolution from clinical to molecular approaches. Nat. Med..

[5] Comité Ejecutivo de la GEMA (2015). Guía Española Para el Manejo del Asma. LUZÁN 5.

[6] Cisneros Serrano C., Melero Moreno C., Almonacid Sánchez C., Perpiñá Tordera M., Picado Valles C., Martínez Moragón E., de Llano L.P., Soto Campos J.G., Urrutia Landa I., García Hernández G. (2015). Guidelines for severe uncontrolled asthma. Arch. Bronconeumol..

[7] Papadopoulos N.G., Arakawa H., Carlsen K.-H., Custovic A., Gern J., Lemanske R., le Souef P., Makela M., Roberts G., Wong G. (2012). International consensus on (ICON) pediatric asthma. Allergy.

[8] Lötvall J., Akdis C.A., Bacharier L.B., Bjermer L., Casale T.B., Custovic A., Lemanske R.F., Wardlaw A.J., Wenzel S.E., Greenberger P.A. (2011). Asthma endotypes: A new approach to classification of disease entities within the asthma syndrome. J. Allergy Clin. Immunol..

[9] Asher M.I., Montefort S., Björkstén B., Lai C.K.W., Strachan D.P., Weiland S.K., ISAAC Phase Three Study Group (2006). Worldwide time trends in the prevalence of symptoms of asthma, allergic rhinoconjunctivitis, and eczema in childhood: ISAAC phases one and three repeat multicountry cross-sectional surveys. Lancet.

[10] Björkstén B., Clayton T., Ellwood P., Stewart A., Strachan D., ISAAC Phase III Study Group (2008). Worldwide time trends for symptoms of rhinitis and conjunctivitis: Phase III of the International Study of Asthma and Allergies in Childhood. Pediatr. Allergy Immunol..

[11] Yuksel H., Dinc G., Sakar A., Yilmaz O., Yorgancioglu A., Celik P., Ozcan C. (2008). Prevalence and comorbidity of allergic eczema, rhinitis, and asthma in a city in western Turkey. J. Investig. Allergol. Clin. Immunol..

[12] Almqvist C., Lundholm C. (2015). Population-based data on asthma and allergic disease call for advanced epidemiologic methods. J. Allergy Clin. Immunol..

[13] Isolauri E., Huurre A., Salminen S., Impivaara O. (2004). The allergy epidemic extends beyond the past few decades. Clin. Exp. Allergy.

[14] Chang C. (2012). Asthma in children and adolescents: A comprehensive approach to diagnosis and management. Clin. Rev. Allergy Immunol..

[15] Mallol J., Crane J., von Mutius E., Odhiambo J., Keil U., Stewart A., ISAAC Phase Three Study Group (2013). The International Study of Asthma and Allergies in Childhood (ISAAC) Phase Three: A global synthesis. Allergol. Immunopathol..

[16] Pawankar R., Canonica G., Holgate S., Lockey R. (2011). World Health Organization (WAO) White Book on Allergy.

[17] Covar R.A., Strunk R., Zeiger R.S., Wilson L.A., Liu A.H., Weiss S., Tonascia J., Spahn J.D., Szefler S.J., Childhood Asthma Management Program Research Group (2010). Predictors of remitting, periodic, and persistent childhood asthma. J. Allergy Clin. Immunol..

[18] Murray C.S., Woodcock A., Langley S.J., Morris J., Custovic A., IFWIN Study Team (2006). Secondary prevention of asthma by the use of Inhaled Fluticasone Propionate in Wheezy INfants (IFWIN): Double-blind, randomised, controlled study. Lancet.

[19] Guilbert T.W., Morgan W.J., Krawiec M., Lemanske R.F., Sorkness C., Szefler S.J., Larsen G., Spahn J.D., Zeiger R.S., Heldt G. (2004). The Prevention of Early Asthma in Kids study: Design, rationale and methods for the Childhood Asthma Research and Education network. Control. Clin. Trials.

[20] Weiss S.T., Horner A., Shapiro G., Sternberg A.L. (2001). Childhood Asthma Management Program (CAMP) Research Group. The prevalence of environmental exposure to perceived asthma triggers in children with mild-to-moderate asthma: Data from the Childhood Asthma Management Program (CAMP). J. Allergy Clin. Immunol..

[21] Accordini S., Corsico A.G., Braggion M., Gerbase M.W., Gislason D., Gulsvik A., Heinrich J., Janson C., Jarvis D., Jõgi R. (2013). The cost of persistent asthma in Europe: An international population-based study in adults. Int. Arch. Allergy Immunol..

[22] Calderon M.A., Demoly P., van Wijk R.G., Bousquet J., Sheikh A., Frew A., Scadding G., Bachert C., Malling H.J., Valenta R. (2012). EAACI: A European Declaration on Immunotherapy. Designing the future of *allergen* specific immunotherapy. Clin. Transl. Allergy.

[23] Heineke M.H., van Egmond M. (2017). Immunoglobulin A: Magic bullet or Trojan horse?. Eur. J. Clin. Investig..

[24] Chen K., Cerutti A. (2011). The function and regulation of immunoglobulin D. Curr. Opin. Immunol..

[25] Rigante D. (2016). The truth on IgD in the ploy of immune surveillance and inflammation. Immunol. Res..

[26] Kelly B.T., Grayson M.H. (2016). Immunoglobulin E, what is it good for?. Ann. Allergy Asthma Immunol..

[27] Aschermann S., Lux A., Baerenwaldt A., Biburger M., Nimmerjahn F. (2010). The other side of immunoglobulin G: Suppressor of inflammation. Clin. Exp. Immunol..

[28] Manson J.J., Mauri C., Ehrenstein M.R. (2005). Natural serum IgM maintains immunological homeostasis and prevents autoimmunity. Springer Semin. Immunopathol..

[29] Matondang C.S. (1991). Spectrum of asthma in children visiting the outpatient clinic of the subdivision of allergy and immunology. Paediatr. Indones..

[30] McNichol K.N., Williams H.E. (1973). Spectrum of asthma in children. II. Allergic components. Br. Med. J..

[31] Kotaniemi-Syrjänen A., Reijonen T.M., Romppanen J., Korhonen K., Savolainen K., Korppi M. (2003). Allergen-specific immunoglobulin E antibodies in wheezing infants: The risk for asthma in later childhood. Pediatrics.

[32] Resch Y., Michel S., Kabesch M., Lupinek C., Valenta R., Vrtala S. (2015). Different IgE recognition of mite allergen components in asthmatic and nonasthmatic children. J. Allergy Clin. Immunol..

[33] Buendía E., Zakzuk J., Mercado D., Alvarez A., Caraballo L. (2015). The IgE response to Ascaris molecular components is associated with clinical indicators of asthma severity. World Allergy Organ. J..

[34] Naqvi M., Choudhry S., Tsai H.-J., Thyne S., Navarro D., Nazario S., Rodriguez-Santana J.R., Casal J., Torres A., Chapela R. (2007). Association between IgE levels and asthma severity among African American, Mexican, and Puerto Rican patients with asthma. J. Allergy Clin. Immunol..

[35] Carroll W.D., Lenney W., Child F., Strange R.C., Jones P.W., Whyte M.K., Primhak R.A., Fryer A.A. (2006). Asthma severity and atopy: How clear is the relationship?. Arch. Dis. Child..

[36] Warm K., Hedman L., Lindberg A., Lötvall J., Lundbäck B., Rönmark E. (2015). Allergic sensitization is age-dependently associated with rhinitis, but less so with asthma. J. Allergy Clin. Immunol..

[37] Siroux V., Oryszczyn M.-P., Paty E., Kauffmann F., Pison C., Vervloet D., Pin I. (2003). Relationships of allergic sensitization, total immunoglobulin E and blood eosinophils to asthma severity in children of the EGEA Study. Clin. Exp. Allergy.

[38] Tanaka A., Jinno M., Hirai K., Miyata Y., Mizuma H., Yamaguchi M., Ohta S., Watanabe Y., Yamamoto M., Suzuki S. (2014). Longitudinal increase in total IgE levels in patients with adult asthma: An association with poor asthma control. Respir. Res..

[39] Maneechotesuwan K., Sujaritwongsanon P., Suthamsmai T. (2010). IgE production in allergic asthmatic patients with different asthma control status. J. Med. Assoc. Thail..

[40] Lowe P.J., Tannenbaum S., Gautier A., Jimenez P. (2009). Relationship between omalizumab pharmacokinetics, IgE pharmacodynamics and symptoms in patients with severe persistent allergic (IgE-mediated) asthma. Br. J. Clin. Pharmacol..

[41] Kulus M., Hébert J., Garcia E., Fowler Taylor A., Fernandez Vidaurre C., Blogg M. (2010). Omalizumab in children with inadequately controlled severe allergic (IgE-mediated) asthma. Curr. Med. Res. Opin..

[42] Pereira Santos M.C., Campos Melo A., Caetano A., Caiado J., Mendes A., Pereira Barbosa M., Branco Ferreira M. (2015). Longitudinal study of the expression of FcεRI and IgE on basophils and dendritic cells in association with basophil function in two patients with severe allergic asthma treated with Omalizumab. Eur. Ann. Allergy Clin. Immunol..

[43] Swain S.L., Weinberg A.D., English M., Huston G. (1990). IL-4 directs the development of Th2-like helper effectors. J. Immunol..

[44] Noben-Trauth N., Hu-Li J., Paul W.E. (2000). Conventional, naive CD4+ T cells provide an initial source of IL-4 during Th2 differentiation. J. Immunol..

[45] Paul W.E., Zhu J. (2010). How are TH2-type immune responses initiated and amplified?. Nat. Rev. Immunol..

[46] Wu L.C., Zarrin A.A. (2014). The production and regulation of IgE by the immune system. Nat. Rev. Immunol..

[47] Looney T.J., Lee J.-Y., Roskin K.M., Hoh R.A., King J., Glanville J., Liu Y., Pham T.D., Dekker C.L., Davis M.M. (2016). Human B-cell isotype switching origins of IgE. J. Allergy Clin. Immunol..

[48] Robertson D.G., Kerigan A.T., Hargreave F.E., Chalmers R., Dolovich J. (1974). Late asthmatic responses induced by ragweed pollen allergen. J. Allergy Clin. Immunol..

[49] O’Byrne P. (1998). Asthma pathogenesis and allergen-induced late responses. J. Allergy Clin. Immunol..

[50] Roth M., Zhong J., Zumkeller C., S’ng C.T., Goulet S., Tamm M. (2013). The role of IgE-receptors in IgE-dependent airway smooth muscle cell remodelling. PLoS ONE.

[51] Redhu N.S., Shan L., Al-Subait D., Ashdown H.L., Movassagh H., Lamkhioued B., Gounni A.S. (2013). IgE induces proliferation in human airway smooth muscle cells: Role of MAPK and STAT3 pathways. Allergy Asthma Clin. Immunol..

[52] Janeway C., Travers P., Walport M., Shlomchik M. (2001). Immunobiology: The Immune System in Health and Disease.

[53] Hart P.H. (2001). Regulation of the inflammatory response in asthma by mast cell products. Immunol. Cell Biol..

[54] Amin K. (2012). The role of mast cells in allergic inflammation. Respir. Med..

[55] Theoharides T.C., Alysandratos K.-D., Angelidou A., Delivanis D.-A., Sismanopoulos N., Zhang B., Asadi S., Vasiadi M., Weng Z., Miniati A. (2012). Mast cells and inflammation. Biochim. Biophys. Acta.

[56] Skokos D., Botros H.G., Demeure C., Morin J., Peronet R., Birkenmeier G., Boudaly S., Mécheri S. (2003). Mast cell-derived exosomes induce phenotypic and functional maturation of dendritic cells and elicit specific immune responses in vivo. J. Immunol..

[57] Brown J.M., Wilson T.M., Metcalfe D.D. (2008). The mast cell and allergic diseases: Role in pathogenesis and implications for therapy. Clin. Exp. Allergy.

[58] Wills-Karp M., Luyimbazi J., Xu X., Schofield B., Neben T.Y., Karp C.L., Donaldson D.D. (1998). Interleukin-13: Central mediator of allergic asthma. Science.

[59] Galli S.J., Tsai M. (2012). IgE and mast cells in allergic disease. Nat. Med..

[60] D’Amato G., Piccolo A., Salzillo A., Noschese P., D’Amato M., Liccardi G. (2007). A recombinant humanized anti-IgE monoclonal antibody (omalizumab) in the therapy of moderate-to-severe allergic asthma. Recent Pat. Inflamm. Allergy Drug Discov..

[61] D’Amato G., Liccardi G., Noschese P., Salzillo A., D’Amato M., Cazzola M. (2004). Anti-IgE monoclonal antibody (omalizumab) in the treatment of atopic asthma and allergic respiratory diseases. Curr. Drug Targets Inflamm. Allergy.

[62] Licari A., Marseglia G., Castagnoli R., Marseglia A., Ciprandi G. (2015). The discovery and development of omalizumab for the treatment of asthma. Expert Opin. Drug Discov..

[63] (2002). Omalizumab: Anti-IgE monoclonal antibody E25, E25, humanised anti-IgE MAb, IGE 025, monoclonal antibody E25, Olizumab, Xolair, rhuMAb-E25. BioDrugs.

[64] Boulet L.P., Chapman K.R., Côté J., Kalra S., Bhagat R., Swystun V.A., Laviolette M., Cleland L.D., Deschesnes F., Su J.Q. (1997). Inhibitory effects of an anti-IgE antibody E25 on allergen-induced early asthmatic response. Am. J. Respir. Crit. Care Med..

[65] Massanari M., Holgate S.T., Busse W.W., Jimenez P., Kianifard F., Zeldin R. (2010). Effect of omalizumab on peripheral blood eosinophilia in allergic asthma. Respir. Med..

[66] Chipps B.E., Marshik P.L. (2004). Targeted IgE therapy for patients with moderate to severe asthma. Biotechnol. Healthc..

[67] Boyman O., Kaegi C., Akdis M., Bavbek S., Bossios A., Chatzipetrou A., Eiwegger T., Firinu D., Harr T., Knol E. (2015). EAACI IG Biologicals task force paper on the use of biologic agents in allergic disorders. Allergy.

[68] Humbert M., Busse W., Hanania N.A., Lowe P.J., Canvin J., Erpenbeck V.J., Holgate S. (2014). Omalizumab in asthma: An update on recent developments. J. Allergy Clin. Immunol. Pract..

[69] Levine S.J., Wenzel S.E. (2010). Narrative review: The role of Th2 immune pathway modulation in the treatment of severe asthma and its phenotypes. Ann. Intern. Med..

[70] Fajt M.L., Wenzel S.E. (2015). Asthma phenotypes and the use of biologic medications in asthma and allergic disease: The next steps toward personalized care. J. Allergy Clin. Immunol..

[71] Rudulier C.D., Larché M., Moldaver D. (2016). Treatment with anti-cytokine monoclonal antibodies can potentiate the target cytokine rather than neutralize its activity. Allergy.

[72] Roth M., Tamm M. (2010). The effects of omalizumab on IgE-induced cytokine synthesis by asthmatic airway smooth muscle cells. Ann. Allergy Asthma Immunol..

[73] Takaku Y., Soma T., Nishihara F., Nakagome K., Kobayashi T., Hagiwara K., Kanazawa M., Nagata M. (2013). Omalizumab attenuates airway inflammation and interleukin-5 production by mononuclear cells in patients with severe allergic asthma. Int. Arch. Allergy Immunol..

[74] Domingo C. (2014). Omalizumab for severe asthma: Efficacy beyond the atopic patient?. Drugs.

[75] Hill D.A., Siracusa M.C., Ruymann K.R., Tait Wojno E.D., Artis D., Spergel J.M. (2014). Omalizumab therapy is associated with reduced circulating basophil populations in asthmatic children. Allergy.

[76] Sharquie I.K., Al-Ghouleh A., Fitton P., Clark M.R., Armour K.L., Sewell H.F., Shakib F., Ghaemmaghami A.M. (2013). An investigation into IgE-facilitated allergen recognition and presentation by human dendritic cells. BMC Immunol..

[77] Gill M.A., Bajwa G., George T.A., Dong C.C., Dougherty I.I., Jiang N., Gan V.N., Gruchalla R.S. (2010). Counterregulation between the FcepsilonRI pathway and antiviral responses in human plasmacytoid dendritic cells. J. Immunol..

[78] Lommatzsch M., Korn S., Buhl R., Virchow J.C. (2014). Against all odds: Anti-IgE for intrinsic asthma?. Thorax.

[79] Busse W.W., Morgan W.J., Gergen P.J., Mitchell H.E., Gern J.E., Liu A.H., Gruchalla R.S., Kattan M., Teach S.J., Pongracic J.A. (2011). Randomized trial of omalizumab (anti-IgE) for asthma in inner-city children. N. Engl. J. Med..

[80] Teach S.J., Gill M.A., Togias A., Sorkness C.A., Arbes S.J., Calatroni A., Wildfire J.J., Gergen P.J., Cohen R.T., Pongracic J.A. (2015). Preseasonal treatment with either omalizumab or an inhaled corticosteroid boost to prevent fall asthma exacerbations. J. Allergy Clin. Immunol..

[81] Ortega H.G., Liu M.C., Pavord I.D., Brusselle G.G., FitzGerald J.M., Chetta A., Humbert M., Katz L.E., Keene O.N., Yancey S.W. (2014). Mepolizumab treatment in patients with severe eosinophilic asthma. N. Engl. J. Med..

[82] Bel E.H., Wenzel S.E., Thompson P.J., Prazma C.M., Keene O.N., Yancey S.W., Ortega H.G., Pavord I.D. (2014). Oral glucocorticoid-sparing effect of mepolizumab in eosinophilic asthma. N. Engl. J. Med..

[83] Pavord I.D., Korn S., Howarth P., Bleecker E.R., Buhl R., Keene O.N., Ortega H., Chanez P. (2012). Mepolizumab for severe eosinophilic asthma (DREAM): A multicentre, double-blind, placebo-controlled trial. Lancet.

[84] Castro M., Zangrilli J., Wechsler M.E., Bateman E.D., Brusselle G.G., Bardin P., Murphy K., Maspero J.F., O’Brien C., Korn S. (2015). Reslizumab for inadequately controlled asthma with elevated blood eosinophil counts: Results from two multicentre, parallel, double-blind, randomised, placebo-controlled, phase 3 trials. Lancet Respir. Med..

[85] Bleecker E.R., FitzGerald J.M., Chanez P., Papi A., Weinstein S.F., Barker P., Sproule S., Gilmartin G., Aurivillius M., Werkström V. (2016). Efficacy and safety of benralizumab for patients with severe asthma uncontrolled with high-dosage inhaled corticosteroids and long-acting β2-agonists (SIROCCO): A randomised, multicentre, placebo-controlled phase 3 trial. Lancet.

[86] FitzGerald J.M., Bleecker E.R., Nair P., Korn S., Ohta K., Lommatzsch M., Ferguson G.T., Busse W.W., Barker P., Sproule S. (2016). Benralizumab, an anti-interleukin-5 receptor α monoclonal antibody, as add-on treatment for patients with severe, uncontrolled, eosinophilic asthma (CALIMA): A randomised, double-blind, placebo-controlled phase 3 trial. Lancet.

[87] Wenzel S., Castro M., Corren J., Maspero J., Wang L., Zhang B., Pirozzi G., Sutherland E.R., Evans R.R., Joish V.N. (2016). Dupilumab efficacy and safety in adults with uncontrolled persistent asthma despite use of medium-to-high-dose inhaled corticosteroids plus a long-acting β2 agonist: A randomised double-blind placebo-controlled pivotal phase 2b dose-ranging trial. Lancet.

[88] Solèr M., Matz J., Townley R., Buhl R., O’Brien J., Fox H., Thirlwell J., Gupta N., della Cioppa G. (2001). The anti-IgE antibody omalizumab reduces exacerbations and steroid requirement in allergic asthmatics. Eur. Respir. J..

[89] Buhl R., Hanf G., Solèr M., Bensch G., Wolfe J., Everhard F., Champain K., Fox H., Thirlwell J. (2002). The anti-IgE antibody omalizumab improves asthma-related quality of life in patients with allergic asthma. Eur. Respir. J..

[90] Finn A., Gross G., van Bavel J., Lee T., Windom H., Everhard F., Fowler-Taylor A., Liu J., Gupta N. (2003). Omalizumab improves asthma-related quality of life in patients with severe allergic asthma. J. Allergy Clin. Immunol..

[91] Corren J., Casale T., Deniz Y., Ashby M. (2003). Omalizumab, a recombinant humanized anti-IgE antibody, reduces asthma-related emergency room visits and hospitalizations in patients with allergic asthma. J. Allergy Clin. Immunol..

[92] Bousquet J., Cabrera P., Berkman N., Buhl R., Holgate S., Wenzel S., Fox H., Hedgecock S., Blogg M., Cioppa G.D. (2005). The effect of treatment with omalizumab, an anti-IgE antibody, on asthma exacerbations and emergency medical visits in patients with severe persistent asthma. Allergy.

